# Role for TRPA1 receptor channels in trigeminal afferent activation and neuropeptide release from rat cranial dura mater

**DOI:** 10.1186/1129-2377-14-S1-P70

**Published:** 2013-02-21

**Authors:** S Albrecht, AC Denner, M Eberhardt, R DeCol, KB Messlinger

**Affiliations:** 1 Institute of Physiology & Pathophysiology, University of Erlangen-Nuernberg, Germany

## Introduction

TRPA1 receptor channels are activated by environmental irritants and by endogenous mediators released during inflammatory conditions ( McMahon & Wood 2006 ). Activation of TRPA1 receptors causes CGRP release from trigeminal ganglion neurons and increases meningeal blood flow upon nasal stimulation ( Kunkler et al. 2011 ), providing evidence that TRPA1 receptors may be involved in the generation of headaches.

## Objective

To further examine the role of TRPA1 receptor activation in processes presumably associated with headache generation, we investigated the effects of the TRPA1 agonist acrolein on functions involved in meningeal nociception using four different rat models.

## Methods

The discharge activity of single meningeal afferents innervating the dura mater was recorded in a hemisected cranial preparation. 2. The activity of second order neurons in the spinal trigeminal nucleus with meningeal afferent input was recorded in anaesthetised animals. 3. In the hemisected cranial preparation the dura mater was superfused with synthetic interstitial fluid, and stimulated calcitonin gene-related peptide (CGRP) release was measured using an ELISA. 4. Meningeal blood flow was monitored in the exposed dura mater of anaesthetised animals using laser Doppler flowmetry. In all preparations the dura mater was stimulated with the TRPA1 agonist acrolein (10-4 M).

## Results

Acrolein did not elicit discharges in meningeal Aδ- or C-fibres in the hemisected cranial preparation and did not change the discharge activity of second order neurons with meningeal receptive fields in anaesthetized animals. In contrast, acrolein significantly stimulated CGRP release from the dura mater within 5 min and increased meningeal blood flow. Both responses were suppressed by the TRPA1 inhibitor HC030031.

## Conclusion

TRPA1 channel activation causes neuropeptide release from meningeal afferents but does not generate propagated afferent information. Therefore an important role for peripheral TRPA1 receptors in headache generation appears unlikely.
